# Operational method of solution of linear non-integer ordinary and partial differential equations

**DOI:** 10.1186/s40064-016-1734-3

**Published:** 2016-02-09

**Authors:** K. V. Zhukovsky

**Affiliations:** Faculty of Physics, M.V. Lomonosov Moscow State University, Leninskie Gory, Moscow, Russia 119991

**Keywords:** Inverse operator, Derivative, Differential equation, Special functions, Hermite and Laguerre polynomials, 02.30 Gp, Hq, Jr, Mv, Nw, Tb, Uu, Vv, Zz, 41.85.Ja, 03.65.Db, 05.60.Cd

## Abstract

We propose operational method with recourse to generalized forms of orthogonal polynomials for solution of a variety of differential equations of mathematical physics. Operational definitions of generalized families of orthogonal polynomials are used in this context. Integral transforms and the operational exponent together with some special functions are also employed in the solutions. The examples of solution of physical problems, related to such problems as the heat propagation in various models, evolutional processes, Black–Scholes-like equations etc. are demonstrated by the operational technique.

## Background

Differential equations, besides playing important role in pure mathematics, constitute fundamental part of mathematical description of physical processes. Thus, obtaining the solutions for differential equations is of paramount importance. Few types of differential equations allow explicit and straightforward analytical solutions. The development of computer methods and proper technical means in twenty-first century facilitated equations solving. There are numerous numerical methods for solving differential equations (see, for example, Von Rosenberg 1969; Smith 1985; Ghia et al. 1982; Ames 2014; Johnson 2012; Carnahan et al. 1969). However, understanding of the obtained solutions and of the interplay of various parameters in them can be best done in analytical form. Thus, despite the revolutionary breakthrough in numerical calculus, analytical studies remain requested. Exact and approximate solutions are searched, while the first are certainly preferred. Recently some fractional type ordinary and partial differential equations involving non-integer derivatives were explored in Demiray et al. (2015). Exact analytical solutions for such equations were obtained as sums of a vector-type functional (Akinlar and Kurulay 2013). Some partial differential–algebraic equations were also solved by the power series method (Filobello-Nino et al. 2015; Benhammouda and Vazquez-Leal 2014). The solutions were obtained in the form of converging series. Fokker–Planck type equations were recently solved by differential transforms in Hesam et al. (2012); the solutions obtained in form of the form of rapidly convergent series. Semi-analytical techniques for the solution of differential–algebraic equations were developed (Soltanian et al. 2013) and applied for description of an incompressible viscous fluid flow. Approximate solutions for some nonlinear delay differential equations were obtained (Caruntu and Bota 2014) and applied to a biologic model. A modified method of simplest equation was proposed in Vitanov et al. (2015) to find exact analytical solutions of nonlinear partial differential equations. In many cases, these solutions are best formulated in terms of special functions and orthogonal polynomials when used for relevant models of physical processes. Hyperbolic, elliptic Weierstrass and Jacobi type, generalized Airy and Bessel type functions are used (Vitanov et al. 2015; Dattoli et al. 2008, 2009; Appèl and Kampé de Fériet 1926; Dattoli 2000; Dattoli et al. 2005; Zhukovsky 2014, 2015a, b, c, d); expansion in series of Hermite and Laguerre polynomials (Appèl and Kampé de Fériet 1926) are employed. These polynomials possess generalized forms with many variables and indices (Dattoli 2000; Dattoli et al. 2005). In this framework the operational definitions for the polynomials are useful (Erdélyi et al. 1953). The above mentioned recent developments in analytical and semi-analytical equations solutions (Demiray et al. 2015; Akinlar and Kurulay 2013; Filobello-Nino et al. 2015; Benhammouda and Vazquez-Leal 2014; Hesam et al. 2012; Soltanian et al. 2013; Caruntu and Bota 2014; Vitanov et al. 2015) are indeed capable of reducing the size of computational work.

In what follows we shall demonstrate the abilities of the operational approach for solution of differential equations. With the help of this general method we will obtain exact analytical solutions for a broad class of differential equations, including those with non-integer derivatives, evolution type equations, generalized forms of heat, mass transfer and Black–Scholes type equations, involving also the Laguerre derivative operator. Recently, this method was applied for solution of some differential equations in Zhukovsky (2014, 2015a) and Dattoli et al. (2007). These equations cover a broad range of physical problems: from propagation and radiation of accelerated charges to heat and mass transfer (see, for example, Haimo and Markett 1992a, b; Zhukovsky 2016). Operational exponent, employed for solution, finds its application even for description of such fundamentals of nature as quarks and neutrinos (Dattoli and Zhukovsky 2007a, b, 2008). We will not include error analysis in our work since the proposed operational method produces analytical solutions, which satisfy the equations exactly.

When it comes to a numerical analysis, there are also practical and theoretical reasons for examining the process of inverting differential operators. Indeed, the inverse or integral form of a differential equation displays explicitly the input–output relationship of the system. Moreover, integral operators are computationally and theoretically less troublesome than differential operators; for example, differentiation emphasizes data errors, whereas integration averages them. Thus, it may be advantageous to apply computational procedures to differential systems, based on the inverse or integral description of the system.

The evident concept of an inverse function is a function that undoes another function: if an input *x* into the function *ƒ* produces an output *y*, then putting *y* into the inverse function *g* produces the output *x*, and vice versa. i.e.,$f(x) = y$ and $g(y) = x$ or $g(f(x)) = x$. If a function ƒ has an inverse *ƒ*^−1^, it is invertible and the inverse function is then uniquely determined by ƒ. We can develop similar approach with regard to differential operators. In what follows we will further develop this technique and explore its relation with extended forms of orthogonal polynomials, producing useful relations for solution of a variety of differential equations, by means of inverse derivative. The relevant physical problems will be considered.

Let us denote a common differential operator $D = {d} / {dx}$. The inverse derivative of a function *ƒ*(*x*) is another function *F*(*x*): $D^{ - 1} f(x) = F(x)$, whose derivative is $F^{\prime}(x) = f(x)$. Naturally, we expect anti-derivative or inverse derivative $D^{ - 1}$ as the inverse operation of differentiation to be an integral operator. The generalized form of the inverse derivative of *ƒ*(*x*) with respect to *x* evidently is $\int {f(x) = F(x) + C}$, where *C*—the constant of integration. The action of the inverse derivative operator of the *n*-th order

1 $$ D_{x}^{ - n} f(x) = \frac{1}{(n - 1)!}\int\limits_{0}^{x} {(x - \xi )^{n - 1} } f(\xi )d\xi ,\quad (n \in N = \{ 1,2,3, \ldots \} ) $$

can be complemented with the definition for its zeros order action as follows:

2 $$ D_{x}^{0} f(x) = f(x). $$

Hence, we can write:

3 $$ D_{x}^{ - n} 1 = \frac{{x^{n} }}{n!},\quad (n \in N_{0} = N \cup \{ 0\} ). $$

For a general form of differential equation

4 $$ \psi (D)F(x) = f(x), $$

where $\psi (D)$—differential operator, composed of derivatives or various orders: *D*, *D*^2^, …, *D*^n^ the inverse differential operator $1/\psi (D)$ or $(\psi (D))^{ - 1}$ is defined, such that

5 $$ \psi (D)(\psi (D))^{ - 1} f(x) = f(x). $$

From (4) we obtain the particular integral

6 $$ F(x) = (\psi (D))^{ - 1} f(x) $$

The inverse differential operator $(\psi (D))^{ - 1}$ is evidently linear, i.e.

7 $$ \frac{1}{\psi (D)}\left\{ {af(x) + bg(x)} \right\} = a\frac{1}{\psi (D)}f(x) + b\frac{1}{\psi (D)}g(x), $$

where *a* and *b* are constants, *ƒ*(*x*) and *g*(*x*) are some functions of *x*. Let us consider an elementary example of the following simple equation:

8 $$ \psi (D)F(x) = e^{\alpha x} , $$

where $\psi (D)$ consists of derivatives of various orders. The action of $\psi (D)$ on exp(*αx*) results in $\psi (D)e^{\alpha x} = (c_{n} D^{n} + \cdots + c_{1} D + c_{0} )e^{\alpha x} = \psi (\alpha )e^{\alpha x}$; applying inverse operator $(\hat{\psi }(D))^{ - 1}$ to both sides, we obtain: $e^{ax} = \psi (\alpha )\frac{1}{\psi (D)}e^{\alpha x}$ or $\frac{{e^{ax} }}{\psi (\alpha )} = \frac{1}{\psi (D)}e^{\alpha x}$ and we conclude that Eq. (8) possesses the following particular integral:

9 $$ F(x) = (\psi (D))^{ - 1} e^{\alpha x} = \frac{{e^{\alpha x} }}{\psi (\alpha )}. $$

It can be easily shown by means of the inverse derivative operator that (9), being the solution of Eq. (8), is true also for the operator $\psi (D)$ with higher than the second order derivatives. Moreover, it is easy to prove the following identity:

10 $$ (\psi (D))^{ - 1} e^{\alpha x} f(x) = e^{\alpha x} (\psi (D + \alpha ))^{ - 1} f(x), $$

and the action of the inverse operator $(\psi (D + \alpha ))^{ - 1}$ on a function ƒ, which can be expressed via the inverse differential operator $(\psi (D))^{ - 1}$, reads as follows:

11 $$ F(x) = (\psi (D + \alpha ))^{ - 1} f(x) = e^{ - \alpha x} (\psi (D))^{ - 1} e^{\alpha x} f(x). $$

### Inverse differential and exponential operators for solution of some non-integer ordinary differential equations

Let us consider the following equation:

12 $$ \left( {\beta^{2} - (D + \alpha )^{2} } \right)^{\nu } F(x) = f(x),\quad D + \alpha \equiv \tilde{D}, $$

where we denote operator $\tilde{D} \equiv D + \alpha$ and *α*, *β*—constants. In order to find the particular integral

13 $$ F(x) = \left( {\beta^{2} - \tilde{D}^{2} } \right)^{ - \nu } f(x) $$

we shall make use of the well-known operational identity (Erdélyi et al. 1953; Srivastava and Manocha 1984), frequently used in fractional derivative calculus:

14 $$ \hat{q}^{ - \nu } = \frac{1}{\varGamma (\nu )}\int\limits_{0}^{\infty } {\exp ( - \hat{q}t)t^{\nu - 1} dt} ,\quad \hbox{min} \{ \text{Re} (q),\text{Re} (\nu )\} > 0, $$

which reads for the operator $\hat{q} = \beta^{2} - \tilde{D}^{2}$:

15 $$ \left( {\beta^{2} - \tilde{D}^{2} } \right)^{ - \nu } f\left( x \right) = \frac{1}{\varGamma \left( \nu \right)}\int\limits_{0}^{\infty } {\exp ( - \beta^{2} t)t^{\nu - 1} \exp (t\tilde{D}^{2} )f\left( x \right)dt} . $$

There are several ways to proceed with the solution. One of them consists in the following. Note that differential operator $- t\tilde{D}^{2}$ in the exponential reduces to the first order derivative with the help of the following integral presentation for the exponential of a square of an operator $\hat{p}$ (Wolf 1979):

16 $$ \exp \left( {\hat{p}^{2} } \right) = \frac{1}{\sqrt \pi }\int\limits_{ - \infty }^{\infty } {\exp \left( { - \xi^{2} + 2\xi \hat{p}} \right)d} \xi , $$

where $\hat{p} = \sqrt t \tilde{D}$ in our case. Thus, the above formula reads as follows:

17 $$ \exp (t\tilde{D}^{2} )f(x) = \frac{1}{\sqrt \pi }\int\limits_{ - \infty }^{\infty } {\exp ( - \xi^{2} + 2\xi \sqrt t \tilde{D})f(x)d\xi } . $$

Now, if we take into account the action of the operator of translation $\exp (\eta \tilde{D})$ for $\tilde{D} = D + \alpha$:

18 $$ \exp (\eta (D + \alpha ))f(x) = \exp (\eta \alpha )f(x + \eta ), $$

the above-sketched operational procedure yields the following expression for the particular integral (13):

19 $$ F(x) = \frac{1}{\sqrt \pi \varGamma \left( \nu \right)}\int\limits_{0}^{\infty } {t^{\nu - 1} \exp \left( {(\alpha^{2} - \beta^{2} )t} \right)\int\limits_{ - \infty }^{\infty } {\exp \left( { - (\xi - \sqrt t \alpha )^{2} } \right)f(x + 2\xi \sqrt t )d\xi } dt} . $$

Upon performing the change of variables, given by

20 $$ \eta = x + 2\xi \sqrt t \quad {\text{and}}\quad t = \tau^{2} , $$

we finally obtain the solution of the Eq. (12):

21 $$ F(x) = \frac{1}{\sqrt \pi \varGamma \left( \nu \right)}\int\limits_{0}^{\infty } {\tau^{2(\nu - 1)} \exp \left( { - (\beta \tau )^{2} } \right)\int\limits_{ - \infty }^{\infty } {\exp \left( { - \left( {\frac{\eta - x}{2\tau }} \right)^{2} + \alpha (\eta - x)} \right)f(\eta )d\eta } d\tau } . $$

Equation (12) involves a convolution transform $\phi \left( {x,\tau } \right) = G\left( {x,\tau } \right) \cdot f\left( \eta \right)$ or $\phi = \int\nolimits_{ - \infty }^{\infty } {G\left( {x - \eta } \right)f\left( \eta \right)d\eta }$ with the kernel, equal to the Gauss frequency function $G\left( {x,\tau } \right) = \exp ( - (x/2\tau )^{2}  - \alpha x)$, so that

22 $$ F(x) = \frac{1}{\sqrt \pi \,\varGamma \left( \nu \right)}\int\limits_{0}^{\infty } {\tau^{2(\nu - 1)} \exp \left( { - (\beta \tau )^{2} } \right)\phi \left( {x,\tau } \right)d\tau } = \frac{1}{\sqrt \pi \varGamma \left( \nu \right)}\int\limits_{ - \infty }^{\infty } {e^{{2\nu \tau - \beta^{2} e^{2\tau } }} \phi \left( {x,e^{\tau } } \right)d\tau } . $$

Evidently, for *α* = 0

23 $$ \left( {\beta^{2} - D^{2} } \right)^{\nu } F(x) = f(x) $$

and its solution becomes the particular case of (15) with the substitution $\tilde{D} \to D$:

24 $$ F(x) = \frac{1}{\varGamma \left( \nu \right)}\int\limits_{0}^{\infty } {\exp ( - \beta^{2} t)t^{\nu - 1} \hat{S}\,f\left( x \right)dt} , $$

where the differential operator

25 $$ \hat{S} = \exp (tD_{x}^{2} ) \equiv \exp (tD^{2} ) $$

was thoroughly explored by Srivastava and Manocha (1984). In particular, for $f(x) = \exp ( - x^{2} )$ we can make use of the Gleisher operational rule (Srivastava and Manocha 1984)

26 $$ \hat{S}\,f(x) = \exp \left( {y\frac{{\partial^{2} }}{{\partial x^{2} }}} \right)\exp ( - x^{2} ) = \frac{1}{{\sqrt {1 + 4y} }}\exp \left( { - \frac{{x^{2} }}{1 + 4y}} \right) $$

to obtain the following particular solution:

27 $$ F(x) = \frac{1}{\varGamma (\nu )}\int\limits_{0}^{\infty } {\frac{{\exp ( - \beta^{2} t)t^{\nu - 1} }}{{\sqrt {1 + 4t} }}\exp \left( { - \frac{{x^{2} }}{1 + 4t}} \right)dt.} $$

The other approach to Eq. (12) consists in combining the exponential operator technique, the inverse derivative formalism and the Gauss transform. Indeed, when solving equations with $D + \alpha$, we can write the particular integral based upon the operational rule (11), where

28 $$ \psi^{ - 1} (D) = \left( {\beta^{2} - D^{2} } \right)^{ - \nu } . $$

Proceeding with account for (23), (24) and (25), we compute the result of the action of the operator $\exp (\partial_{x}^{2} )$ on $\exp (\alpha x)g(x)$ with the help of the following chain rule:

29 $$ \exp (y\,\partial_{x}^{2} )\exp (\alpha x)g\left( x \right) = \exp (\alpha \,x)\exp (\alpha^{2} y)\exp (2\alpha \,y\,\partial_{x} )\exp (y\partial_{x}^{2} )g\left( x \right), $$

where *y* and *α* are the parameters. It eventually yields the following solution for Eq. (12):

30 $$ F(x) = \frac{1}{\varGamma \left( \nu \right)}\int\limits_{0}^{\infty } {t^{\nu - 1} \exp \left\{ { - (\beta^{2} - \alpha^{2} )t} \right\}\hat{\varTheta }\hat{S}f(x)dt} , $$

where $\hat{\varTheta }$ is the well-known operator of translation:

31 $$ \hat{\varTheta } = \exp (2\alpha tD_{x} ) \equiv \exp \left( {2\alpha t\frac{\partial }{\partial x}} \right),\quad \hat{\varTheta }f(x) = f(x + 2\alpha t) $$

and operator $\hat{S}$ is encountered in problems, related to heat propagation and defined in (25). Its action can be written in integral form by means of common Gauss transforms:

32 $$ Fi(x,t) \equiv \hat{S} f(x) = \frac{1}{{2\sqrt {\pi t} }}\int\limits_{ - \infty }^{\infty } {\exp \left\{ { - \frac{{(x - \xi )^{2} }}{4t}} \right\}f(\xi )d\xi } . $$

Thus, we conclude that the integrand of the solution (30) of the Eq. (12), apart the phase and the factor, responsible for the equation dimension *ν*, is a result of consequent action of operators of heat propagation $\hat{S}$ and operator of translation $\hat{\varTheta }$ on the function $f(x)$:

33 $$ {\text{F}}(x,t) \equiv \hat{\varTheta }\hat{S}f(x) = {\text{Fi}}(x + 2\alpha t,t) = \frac{1}{{2\sqrt {\pi t} }}\int\limits_{ - \infty }^{\infty } {\exp \left\{ { - \frac{{(x + 2\alpha t - \xi )^{2} }}{4t}} \right\}f(\xi )d\xi } . $$

With these notations we can write the solution as follows:

34 $$ F(x) = \frac{1}{\varGamma \left( \nu \right)}\int\limits_{0}^{\infty } {t^{\nu - 1} \exp \left\{ { - (\beta^{2} - \alpha^{2} )t} \right\}F(x,t)dt} . $$

The solution is best illustrated by the example of a Gaussian function $f(x)$:

35 $$ f(x) = \exp ( - x^{2} ). $$

With the help of the above described operational procedure and on the account of (26) we easily obtain the following solution of Eq. (12) for $f(x) = \exp ( - x^{2} )$ is given by a Gaussian:

36 $$ F(x) = \frac{1}{\varGamma (\nu )}\int\limits_{0}^{\infty } {\frac{{t^{\nu - 1} \exp \left\{ { - (\beta^{2} - \alpha^{2} )t} \right\}}}{{\sqrt {1 + 4t} }}\exp \left\{ { - \frac{{(x + 2\alpha t)^{2} }}{1 + 4t}} \right\}dt} . $$

So far, we have demonstrated on simple examples how the usage of inverse derivative together with operational formalism and, in particular, with exponential operator technique, provide elegant and easy way to find solutions in some classes of differential equations. In what follows we will apply the concept of inverse differential operator to find solutions of more sophisticated problems, expressed by differential equations.

### Operational approach and orthogonal polynomials for solution of some non-integer ordinary differential equations

Despite the traditional presentation of many polynomial families is the expansion in series, they are worth being viewed from operational point of view too. Particularly interesting appears their relation with the exponential operators of derivatives and inverse derivatives and special functions. Recently, Hermite, Laguerre and other polynomial families were reconsidered by means of the operational technique (Dattoli 2000; Dattoli et al. 2005, 2006). Hermite polynomials of two variables are explicitly given by the following operational rule (Dattoli 2000) and the series expansion (Gould and Hopper 1962):

37 $$ H_{n}^{\left( m \right)} \left( {x,y} \right) = \exp \left( {y\frac{{\partial^{m} }}{{\partial x^{m} }}} \right)x^{n} ,\quad H_{n}^{\left( m \right)} \left( {x,y} \right) = n!\sum\limits_{r = 0}^{{\left[ {n/m} \right]}} \frac{{x^{n - mr} y^{r} }}{{\left( {n - mr} \right)!r!}}. $$

Note, that

38 $$ H_{n}^{(1)} \left( {x,y} \right) = \left( {x + y} \right)^{n} \quad {\text{and}}\quad H_{n}^{(2)} \left( {x,y} \right) = H_{n} \left( {x,y} \right), $$

where $H_{n} (x,y)$ are more commonly known Hermite polynomials of two variables

39 $$ H_{n} \left( {x,y} \right) = \exp \left( {y\frac{{\partial^{2} }}{{\partial x^{2} }}} \right)x^{n} ,\quad H_{n} \left( {x,y} \right) = n!\sum\limits_{r = 0}^{{\left[ {n/2} \right]}} \frac{{x^{n - 2r} y^{r} }}{{\left( {n - 2r} \right)!r!}} $$

with the following generating function:

40 $$ \exp \left( {xt + yt^{2} } \right) = \sum\limits_{n = 0}^{\infty } {\frac{{t^{n} }}{n!}H_{n} (x,y)} . $$

They can be reduced to the well-known forms of Hermite polynomials of single variable:

41 $$ H_{n} (x,y) = ( - i)^{n} y^{n/2} H_{n} \left( {\frac{ix}{2\sqrt y }} \right) = i^{n} (2y)^{n/2} He_{n} \left( {\frac{x}{{i\sqrt {2y} }}} \right). $$

Note also the following useful and easy to prove relation (Gould and Hopper 1962) for Hermite polynomials:

42 $$ z^{n} H_{n} (x,y) = H_{n} (xz,yz^{2} ). $$

Laguerre polynomials of two variables can be given by an operational relation (Dattoli 2000) or a sum as follows:

43 $$ L_{n} \left( {x,y} \right) = \exp \left( { - y\frac{\partial }{\partial x}x\frac{\partial }{\partial x}} \right)\frac{{\mathop {\left( { - x} \right)}\nolimits^{n} }}{n!} = n!\sum\limits_{r = 0}^{n} {\frac{{( - 1)^{r} y^{n - r} x^{r} }}{{(n - r)!\left( {r!} \right)^{2} }}} . $$

They also reduce to polynomials of a single variable (Srivastava and Manocha 1984) as follows:

44 $$ L_{n} (x,y) = y^{n} L_{n} \left( {\frac{x}{y}} \right),\quad L_{n} (x) = y^{ - n} L_{n} (xy,y) = L_{n} (x,1). $$

The introduction of the second variable in Hermite and Laguerre polynomials allows us to consider them as solutions of partial differential equations with proper initial conditions:

45 $$ \partial_{y} L_{n} \left( {x,y} \right) = - \left( {\partial_{x} x\partial_{x} } \right)L_{n} \left( {x,y} \right)\quad {\text{with}}\quad L_{n} \left( {x,0} \right) = \frac{{( - x)^{n} }}{n!} $$

for Laguerre polynomials $L_{n} (x,y)$ and

46 $$ \partial_{y} H_{n} \left( {x,y} \right) = \partial_{x}^{2} H_{n} \left( {x,y} \right)\quad {\text{with}}\quad H_{n} \left( {x,0} \right) = x^{n} $$

for Hermite polynomials $H_{n} (x,y)$. Importantly, the following differential operators

47 $$ {}_{L}D_{x} = \frac{\partial }{\partial x}x\frac{\partial }{\partial x} = - \hat{P}\quad {\text{and}}\quad \hat{M} = y - D_{x}^{ - 1} $$

are non commutative:

48 $$ [{}_{L}D_{x} ,D_{x}^{ - 1} ] = - 1,\quad ([A,B] = AB - BA) $$

and the following operational relation between them exists (Dattoli et al. 2006):

49 $$ {}_{L}D_{x} = \frac{\partial }{\partial x}x\frac{\partial }{\partial x} = \frac{\partial }{{\partial D_{x}^{ - 1} }}. $$

With the help of this relation, we can extend our approach on differential equations, including operator $\partial_{x} x \partial_{x}$, sometimes called Laguerre derivative ${}_{L}D_{x}$. Then, from the definition (47) we immediately conclude for Laguerre polynomials $L_{n} \left( {x,y} \right)$, defined in (43), that in terms of inverse derivative operator they are expressed as follows:

50 $$ L_{n} (x,y) = n!\sum\limits_{k = 0}^{n} {\frac{{( - x)^{k} y^{n - k} }}{{(n - k)!(k!)^{2} }}} = (y - D_{x}^{ - 1} )^{n} \left\{ 1 \right\}. $$

Moreover, it follows from (43) and (50) that the following operational identity is true for Laguerre polynomials:

51 $$ \exp \left( {\alpha  \frac{\partial }{{\partial D_{x}^{ - 1} }}} \right)L_{n} (x,y) = L_{n} (x,y - \alpha ). $$

Various polynomial families, such as Hermite, Laguerre, Legendre, Shaffer and hybrid polynomials can be reviewed in the context of umbral calculus as members of a more general family of Appèl polynomials, which they belong to. Such consideration is possible in the framework operational approach, where inverse derivative plays important role as an instrument for the study of relevant polynomial families, their features and properties.

For example, let us consider Eq. (12) with $f(x) = x^{k}$. Then, making use of the operational rule (11), and of the identity

52 $$ \exp (yD_{x}^{2} )x^{k} e^{{\alpha  x}} = e^{{(\alpha x + \alpha^{2} y)}} H_{k} (x + 2\alpha y,y), $$

which arises from the operational relation:

53 $$ \exp \left( {y\frac{{\partial^{m} }}{{\partial x^{m} }}} \right)f(x) = f\left( {x + my\frac{{\partial^{m - 1} }}{{\partial x^{m - 1} }}} \right)\left\{ 1 \right\} $$

and from generating function (40), we can write the particular integral (13) for $f(x) = x^{k}$ as follows:

54 $$ F(x) = \left( {\beta^{2} - (D_{x} + \alpha )^{2} } \right)^{ - \nu }  x^{k} = \frac{1}{\varGamma (\nu )}\int\limits_{0}^{\infty } {e^{{ - t((\beta^{2} - \alpha^{2} )}} t^{\nu - 1} H_{k} (x + 2\alpha t,t)dt} . $$

The above expression with the shifted argument of the Hermite polynomial can be derived directly from the general form of the solution (30) and the operational definition of the Hermite polynomials (39). Particular solutions for (12) with $f(x) = x^{k}$ and *α*, *β* = 0, obviously follow from (54).

Note, that even without specifying the type of the function *f* in the r.h.s. of (12) and the values of *ν* and *α* and, we can still disentangle two integrals in (21) by involving Hermite polynomials of two variables (40) as follows:

55 $$ F(x) = \frac{1}{{\sqrt \pi  \varGamma \left( \nu \right)}}\sum\limits_{n = 0}^{\infty } {\int\limits_{0}^{\infty } {\tau^{2(\nu - 1)} \exp ( - \beta^{2} \tau^{2} )H_{n} \left( {\alpha , - \frac{1}{{4\tau^{2} }}} \right)}  d\tau \frac{1}{n!}\int\limits_{ - \infty }^{\infty } {(\eta - x)^{n} f(\eta )d\eta } } . $$

Now, let us consider the following equation:

56 $$ \left( {\beta - \frac{\partial }{\partial x}x\frac{\partial }{\partial x}} \right)^{\nu } F(x) = f(x). $$

From operational point of view, its solution writes as follows:

57 $$ F(x) = \left( {\beta - \frac{\partial }{\partial x}x\frac{\partial }{\partial x}} \right)^{ - \nu } f\left( x \right) = \frac{1}{\varGamma \left( \nu \right)}\int\limits_{0}^{\infty } {\exp ( - \beta  t)t^{\nu - 1} \exp (t {}_{L}D_{x} )f\left( x \right)dt} . $$

Note, that upon the change of variable $t \to e^{t}$ the last integral can be transformed into the following

58 $$ F(x) = \left( {\beta - \frac{\partial }{\partial x}x\frac{\partial }{\partial x}} \right)^{ - \nu } f\left( x \right) = \frac{1}{\varGamma \left( \nu \right)}\int\limits_{ - \infty }^{\infty } {e^{t\nu } e^{{ - \beta  e^{t} }} e^{{e^{t} {}_{L}D_{x} }} f\left( x \right)dt} . $$

In the particular case of $\beta = 1,\;\;\nu = 1$ the above integral presentation reduces to the Laplace transforms for the differential operator ${}_{L}D_{x}$, involved in (57), identical, except for the change ${}_{L}D_{x} \leftrightarrow D_{x}$, to the well known Laplace transforms for the operator $D_{x}$

59 $$ \frac{1}{{1 - \hat{D}_{x} }} = \int\limits_{0}^{\infty } {\exp ( - s(1 - \hat{D}_{x} } ))ds. $$

By choosing $f(x) = x^{n}$ in Eq. (57), we obtain the following result:

60 $$ \left( {\beta - {}_{L}D_{x} } \right)^{ - \nu } x^{n} = \frac{n!}{\varGamma \left( \nu \right)}\int\limits_{0}^{\infty } {\exp ( - \beta  t)t^{n + \nu - 1} L_{n} (x/t)dt} . $$

Moreover, we can write a solution of Eq. (56) for an arbitrary function $f(x)$, if its expansion into series of simple Laguerre polynomials $L_{n} (x)$ exists. Then, provided

61 $$ f(x) = \sum\limits_{n = 0}^{\infty } {c_{n} L_{n} (x)} , $$

and taking into account (51), the solution (57) of Eq. (56) can be also written as the following integral of series of Laguerre polynomials:

62 $$ F(x) = \frac{1}{\varGamma \left( \nu \right)}\int\limits_{0}^{\infty } {\exp ( - \beta  t)t^{\nu - 1} \sum\limits_{n = 0}^{\infty } {c_{n} L_{n} (x,1 - t)} dt} . $$

For the exponential function $f(x) = \exp ( - \gamma  x)$ we can employ the generalized form of the Gleisher operational rule (see Dattoli et al. 2007)

63 $$ \exp ( - t {}_{L}D_{x} ) \cdot \exp ( - \gamma  x) = \frac{1}{{1 - \gamma  t}}\exp \left( { - \frac{{\gamma  x}}{{1 - \gamma  t}}} \right), $$

which immediately yields the following result:

64 $$ \left( {\beta \, - \frac{\partial }{\partial x}x\frac{\partial }{\partial x}} \right)^{ - \nu } \exp ( - \gamma  x) = \frac{1}{\varGamma \left( \nu \right)}\int\limits_{0}^{\infty } {\exp ( - \beta  t)t^{\nu - 1} \frac{1}{{1 + \gamma  t}}\exp \left( { - \frac{{\gamma  x}}{{1 + \gamma  t}}} \right)dt} . $$

Another interesting case arises in the case of the Laguerre derivative ${}_{L}D_{x}$ instead of the common derivative $\partial_{x}$ in the Eq. (12). Let us choose the following initial condition function:

65 $$ f(x) = W_{0} ( - x^{2} ,2), $$

where

66 $$ W_{n} (x,m) = \sum\limits_{s}^{\infty } {\frac{{x^{s} }}{s!(ms + n)!}} , $$

$W_{n} (x,m)$—particular case of the Bessel–Write function (Srivastava and Manocha 1984). Then, following (14), we can write the solution:

67 $$ \left( {\beta^{2} - \left( {\frac{\partial }{\partial x}x\frac{\partial }{\partial x}} \right)^{2} } \right)^{ - \nu } W_{0} ( - x^{2} ,2) = \frac{1}{\varGamma \left( \nu \right)}\int\limits_{0}^{\infty } {\exp ( - \beta^{2}  t)t^{\nu - 1} \exp (t {}_{L}D_{x}^{2} )f\left( x \right)dt} . $$

Eventually, based on the operational definition of Laguerre polynomials (43) and exploiting the other generalized form of the Gleisher operational rule (Dattoli et al. 2006) in the form

68 $$ \exp ({}_{L}D_{x}^{2} )W_{0} ( - x^{2} ,2) = \frac{1}{{\sqrt {1 + 4t} }}W_{0} \left( { - \frac{1}{1 + 4t},2} \right), $$

we obtain:

69 $$ \left( {\beta^{2} - \left( {\frac{\partial }{\partial x}x\frac{\partial }{\partial x}} \right)^{2} } \right)^{ - \nu } W_{0} ( - x^{2} ,2) = \frac{1}{\varGamma \left( \nu \right)}\int\limits_{0}^{\infty } {\exp ( - \beta^{2}  t)t^{\nu - 1} \frac{1}{{\sqrt {1 + 4t} }}W_{0} \left( { - \frac{1}{1 + 4t},2} \right)dt} . $$

Moreover, according to the developed above procedure, we can write the solutions for other than above specified types of equations. For example, we can take advantage of the following generalization of the Laguerre polynomials $L_{n}^{(\alpha )} (x,y)$:

70 $$ L_{n}^{(\alpha )} \left( {x,y} \right) = \exp \left[ { - y\overset{\lower0.5em\hbox{$\smash{\scriptscriptstyle\smile}$}}{D}_{x} } \right]\left\{ {\frac{{\left( { - x} \right)^{n} }}{n!}} \right\}, $$

where operator $$ \overset{\lower0.5em\hbox{$\smash{\scriptscriptstyle\smile}$}}{D}_{x} $$ is defined as follows:

71 $$ \overset{\lower0.5em\hbox{$\smash{\scriptscriptstyle\smile}$}}{D}_{x} = x\partial^{2}_{x} + \left( {\alpha + 1} \right)\partial_{x} . $$

Inverse operator technique easily allows us to write the solution of the equation

72 $$ (x \partial^{2}_{x} + \left( {\alpha + 1} \right)\partial_{x} )^{\nu } F(x) = f(x). $$

Indeed, by following the operational rule (14), we get

73 $$ \overset{\lower0.5em\hbox{$\smash{\scriptscriptstyle\smile}$}}{D}_{x}^{ - \nu } f\left( x \right) = \frac{1}{\varGamma \left( \nu \right)}\int\limits_{0}^{\infty } {\exp ( - \beta  t)t^{\nu - 1} \exp (t \overset{\lower0.5em\hbox{$\smash{\scriptscriptstyle\smile}$}}{D}_{x} )f\left( x \right)dt} $$

and for initial the condition function $f(x) = x^{n}$, we then write:

74 $$ \overset{\lower0.5em\hbox{$\smash{\scriptscriptstyle\smile}$}}{D}_{x}^{ - \nu } x^{n} = \frac{{( - 1)^{n} n!}}{\varGamma \left( \nu \right)}\int\limits_{0}^{\infty } {\exp ( - \beta  t)t^{\nu - 1} L_{n}^{(\alpha )} (x, - t)dt} . $$

Similarly to (60), taking advantage of the generalized form of the Gleisher operational rule from Srivastava and Manocha (1984), we obtain for the operator $$ \overset{\lower0.5em\hbox{$\smash{\scriptscriptstyle\smile}$}}{D}_{x} $$ and $$ f(x) = \exp ( -  \gamma  x) $$:

75 $$ \overset{\lower0.5em\hbox{$\smash{\scriptscriptstyle\smile}$}}{D}_{x}^{ - \nu } \exp ( - \gamma  x) = \frac{1}{\varGamma \left( \nu \right)}\int\limits_{0}^{\infty } {\exp ( - \beta  t)t^{\nu - 1} \frac{1}{{(1 + \gamma  t)^{\alpha + 1} }}\exp \left( { - \frac{{\gamma  x}}{{1 + \gamma  t}}} \right)dt} . $$

### Operational solution of some partial differential equations

The method of the inverse differential operators has multiple applications for solving mathematical problem, describing wide range of physical processes, such as the heat transfer, the diffusion, wave propagation etc. Some of the examples of solution of the heat equation, of the diffusion equation and of their modified forms, the Laguerre heat equation and others, by the inverse derivative method were considered in Dattoli et al. (2006, 2007) and Zhukovsky and Dattoli (2011). In what follows we will explore more complicated, generalized forms of the aforementioned equations, as well as some second order over the time variable partial differential equations will be touched on.

It is worth mentioning that, despite the relation (11) seems trivial to all appearance, it is very useful for solution of a broad family of differential equations by operational method. Indeed, for the differential equation $\psi (D_{x} + \alpha )F(x,t) = f(x,t)$ we can rewrite (11) in the following form: $e^{\alpha x} F(x,t) = \psi^{ - 1} (D_{x} )e^{\alpha x} f(x,t)$ and, for example, for the evolutional type equations, where $f(x,t) = \partial_{t} F(x,t)$, we obtain $\psi (D_{x} )e^{\alpha x} F(x,t) = \partial_{t} e^{\alpha x} F(x,t)$. By denoting $e^{\alpha x} F(x,t) = G(x,t)$ we have the equation $\psi (D_{x} )G(x,t) = \partial_{t} G(x,t)$ with $\psi (D_{x} )$ and with the initial condition $g(x) = G(x,0) = e^{\alpha x} F(x,0) = e^{\alpha x} f(x)$. Thus, in order to obtain the desired solution $F(x,t) = e^{ - \alpha x} G(x,t)$ of the equation $\psi (D_{x} + \alpha )F(x,t) = f(x,t)$ with the initial condition $F(x,0) = f(x)$, we end up with the necessity to solve the equation with $\psi (D_{x} )$ for the function $G(x,t)$ with the initial condition $g(x) = e^{\alpha x} f(x)$. Note that the above discussed method is applicable not only to the evolutional type equations with $\partial_{t}$ in the r.h.s., but also to other operators $\hat{D}\left( t \right)$, acting over the time variable. Indeed, if $G\left( {x,t} \right)$ is the solution of $\psi (D_{x} )G(x,t) = \hat{D}\left( t \right)G(x,t)$ with $g(x) = G(x,0) = e^{\alpha x} F(x,0) = e^{\alpha x} f(x)$, then, following the above scheme, it is easy to demonstrate that $F(x,t) = e^{ - \alpha x} G(x,t)$ is the solution of the equation $\psi (D_{x} + \alpha )F(x,t) = \hat{D}\left( t \right)F(x,t)$ with $F(x,0) = f(x)$. Evidently, in the case of the second order differential operator $\hat{D}\left( t \right)$ the second boundary or initial condition has to be chosen for the differential equation for $F\left( {x,t} \right)$ and, accordingly, for $G\left( {x,t} \right)$. In what follows, we shall apply the above-discussed method to several examples of equations, common in physics and not only.

#### Black–Scholes type equations

To demonstrate the solution of differential equations by the operational method we first consider the following differential equation, which is a generalized form of a Black–Scholes equation, frequently used in financial models:

76 $$ \frac{1}{\rho }\frac{\partial }{\partial t}F\left( {x,t} \right) = \left[ {x^{2} \frac{{\partial^{2} }}{{\partial x^{2} }} + 2\alpha x^{2} \frac{\partial }{\partial x} + \lambda x\frac{\partial }{\partial x} + (\alpha x)^{2} - \mu  } \right]F\left( {x,t} \right),\quad f\left( x \right) = F\left( {x,0} \right), $$

where α, *ρ*, *λ* andи *μ* are the constant coefficients and $f\left( x \right) = F\left( {x,0} \right)$ is the initial condition function. The apparently complicated Eq. (76) reduces to the following form:

77 $$ \frac{1}{\rho }\frac{\partial }{\partial t}G\left( {x,t} \right) = x^{2}  \frac{{\partial^{2} }}{{\partial x^{2} }}G\left( {x,t} \right) + \lambda  x\frac{\partial }{\partial x}G\left( {x,t} \right) - \mu  G\left( {x,t} \right),\quad g\left( x \right) = G\left( {x,0} \right), $$

by the substitution $\partial_{x} \to \partial_{x} + \alpha$. Therefore, according to (11) and to the discussion in the beginning of this chapter, the solution of the Eq. (76) will be found, if we obtain the solution of the Black–Scholes Eq. (77) for $G\left( {x,t} \right)$ with the initial condition function $g\left( x \right) = G\left( {x,0} \right) = e^{\alpha x} F\left( {x,0} \right)$. Then, the solution of (76) reads as follows:

78 $$ F\left( {x,t} \right) = e^{ - \alpha x} G\left( {x,t} \right),\quad {\text{with}}\quad g\left( x \right) = G\left( {x,0} \right) = e^{\alpha x} F\left( {x,0} \right). $$

The Eq. (77) can be easily solved with the help of the operational approach if we distinguish the perfect square of the operator $x \partial_{x}$ (see Dattoli et al. 2007):

79 $$ G\left( {x,t} \right) = \frac{\exp ( - \rho \varepsilon t)}{\sqrt \pi }\int\limits_{ - \infty }^{\infty } {\exp \left[ { - \sigma^{2} + \sigma   \gamma \frac{\lambda }{2\rho }} \right] g \left( {x  \exp (\sigma \gamma )} \right)d\sigma } , $$

where $\gamma = \gamma (t) = 2\sqrt {\rho t}$, $\varepsilon = \mu + \left( {\lambda /2} \right)^{2}$. Let us choose the initial condition $f\left( x \right) = e^{ - \alpha x} x^{n}$ for the Black–Scholes type Eq. (76), i.e. $g(x) = x^{n}$; then the solution (79) has the simple form $G(x,t) = x^{n} \exp \{ \rho  t (n^{2} + \lambda n - \mu )\}$ (see Dattoli et al. 2007) and the Eq. (76) has the following solution:

80 $$ F(x,t) = e^{ - \alpha x} x^{n} \exp \{ \rho  t (n^{2} + \lambda n - \mu )\} . $$

Let us consider another generalization of the Black–Scholes type differential equation with the Laguerre derivative (see 43, 47):

81 $$ \frac{1}{\rho }\frac{\partial }{\partial t}A\left( {x,t} \right) = (\partial_{x} x\partial_{x} )^{2} A\left( {x,t} \right) + \lambda  (\partial_{x} x \partial_{x} )A\left( {x,t} \right) - \mu  A\left( {x,t} \right),\quad g\left( x \right) = A\left( {x,0} \right), $$

where *ρ*, *λ* and *μ* are the constant coefficients and $g\left( x \right) = A\left( {x,t = 0} \right)$ is the initial condition function. The Eq. (81) generalizes and unifies equations of Laguerre diffusion of matter and of heat, considered in Dattoli et al. (2005, 2007). This equation also can be solved by the operational method developed above. Indeed, by distinguishing the perfect square of the Laguerre derivative ${}_{L}D{}_{x} = \partial_{x} x \partial_{x}$ in (81), the solution evidently reads in the form of the exponential $A\left( {x,t} \right) = \exp \left\{ {\rho t\left( {(_{L} D_{x}  + \lambda /2)^{2}  - \varepsilon } \right)} \right\}g(x)$, where $\varepsilon = \mu + \left( {\lambda /2} \right)^{2}$. Now we apply the operational identity (16) to $\exp (a_{L} D_{x} )$ to obtain the following solution for $A(x,t)$:

82 $$ A\left( {x,t} \right) = \frac{{\exp ( - \varepsilon \alpha^{2} )}}{\sqrt \pi }\int\limits_{ - \infty }^{\infty } {\exp \left( { - \sigma^{2} - \sigma  \alpha \lambda - 2\sigma \alpha _{L} D_{x} } \right) g(x)d\sigma } , $$

where $\alpha = \alpha (t) = \sqrt {\rho  t}$. For a given function $g\left( x \right)$ we still have to find the result of the action of the operational exponent $\exp ( - a_{L} D_{x} )g(x)$ and to take the integral $\int {d\sigma }$. Let us choose, for example, the initial condition function $g(x) = ( - x)^{n} /( - x)^{n} n!$. Then we can make use of the operational definition of the Laguerre polynomials (43) and obtain the integral form for $A$

83 $$ A\left( {x,t} \right) = \frac{{\exp ( - \varepsilon \alpha ^{2} /4)}}{{\sqrt \pi  }}\int\limits_{{ - \infty }}^{\infty } {\exp \left( { - \sigma ^{2}  - \sigma \alpha \lambda } \right)L_{n} (x,2\sigma \alpha )d\sigma }. $$

Further integration over $d\sigma$ yields the solution of the Black–Scholes equation with the Laguerre derivative (81) and with the initial condition $g(x) = ( - x)^{n} /n!$ in the following form:

84 $$ A(x,t) = \frac{{\exp ( - \alpha^{2} \mu )}}{\sqrt \pi }n!\sum\limits_{r = 0}^{n} {\frac{{( - x)^{r} (2\alpha )^{n - r} }}{{(n - r)!(r!)^{2} }}} I, $$

where

85 $$ \begin{aligned} I &= \frac{\alpha \lambda }{2}\left( {e^{i(n - r)\pi } - 1} \right)\varGamma \left( {1 + \frac{n - r}{2}} \right)_{1} F_{1} \left( {\frac{1 - (n - r)}{2},\frac{3}{2}, - \left( {\frac{\alpha \lambda }{2}} \right)^{2} } \right) \hfill \\& \quad+ \frac{1}{2}\left( {e^{i(n - r)\pi } + 1} \right)\varGamma \left( {\frac{1 + n - r}{2}} \right)_{1} F_{1} \left( { - \frac{n - r}{2},\frac{1}{2}, - \left( {\frac{\alpha \lambda }{2}} \right)^{2} } \right), \hfill \\ \end{aligned} $$

$\varGamma$ is the gamma function and $_{1} F_{1}$ is the hypergeometric function. Evidently, if the initial condition function can be expanded in the power series of $x$, then the respective solution represents series of the already obtained solution (84). Moreover, if the expansion in series of the Laguerre polynomials for the initial condition function $g(x) = \sum\nolimits_{n} {a_{n} L_{n} (x)}$ exists, then we can exploit the relationships (51) and (44) to obtain the solution in the following form:

86 $$ A\left( {x,t} \right) = \frac{{\exp ( - \varepsilon \alpha ^{2} {\text{/}}4)}}{{\sqrt \pi  }}\sum\limits_{n} {a_{n} \int\limits_{{ - \infty }}^{\infty } {\exp \left( { - \sigma ^{2}  - \sigma \alpha \lambda } \right)L_{n} (x,2\sigma \alpha  + 1)d\sigma } }. $$

In the most general case the solution $A\left( {x,t} \right)$ can be obtained through the following procedure: we employ the operational definitions (47) and the definition of the inverse derivative (1) to write

87 $$ A\left( {x,t} \right) = \frac{{\exp ( - \varepsilon \alpha^{2} )}}{\sqrt \pi }\int\limits_{ - \infty }^{\infty } {\exp \left( { - \sigma^{2} - \sigma  \alpha \lambda } \right)\exp \left( { - 2\sigma \alpha \frac{\partial }{{\partial D_{x}^{ - 1} }}} \right) \phi (D_{x}^{ - 1} )1d\sigma } , $$

where $\phi (D_{x}^{ - 1} )\,{\mathbf{1}} = g(x)$ and the explicit form of the function $\phi$ is given by the integral $\phi (x) = \int_{0}^{\infty } {\exp ( - \kappa )g(x\kappa )d\kappa }$, provided it converges. Note, that $\exp ( - t_{L} D_{x} )g(x)$ is the solution of the Laguerre diffusion equation (Dattoli et al. 2007) $\partial_{t} f(x,t) = -_{L} D_{x} f(x,t)$ with the initial condition $f(x,0) = g(x)$; therefore, the result of the action of the exponential operator in (87) is in fact $f(x,t) = \exp \left( { - t\frac{\partial }{{\partial D_{x}^{ - 1} }}} \right)g(x) = \phi (D_{x}^{ - 1} - t)1$—the solution of the above mentioned Laguerre diffusion equation. Then the desired solution of the Eq. (81) takes the following form:

88 $$ A\left( {x,t} \right) = \frac{{\exp ( - \varepsilon \alpha^{2} )}}{\sqrt \pi }\int\limits_{ - \infty }^{\infty } {\exp \left( { - \sigma^{2} - \sigma  \alpha \lambda } \right)g(x,t) d\sigma } , $$

where

89 $$ g(x,t) = \phi (D_{x}^{ - 1} - 2\sigma \alpha ){\mathbf{1}} = \exp \left( { - 2\sigma \alpha  \frac{\partial }{{\partial D_{x}^{ - 1} }}} \right) \phi (D_{x}^{ - 1} ){\mathbf{1}}\text{.} $$

Consider the following initial condition: $g(x) = W_{0} ( - x^{2} ,2)$, $W_{n} (x,m) = \sum\nolimits_{r = 0}^{\infty } {\frac{{x^{r} }}{r!(mr + n)!}}$, ($m \in {\rm N},\,n \in {\rm N}_{0}$) is the particular case of the Bessel–Write function (Srivastava and Manocha 1984). The corresponding image function is $\phi (x) = \exp ( - x^{2} )$. With account for (16) and (89) we obtain (see also Dattoli et al. 2007)

90 $$ g(x,t) = \frac{1}{\sqrt \pi }\int\limits_{ - \infty }^{\infty } {\exp \left( { - \xi^{2} + 4i\sigma \alpha \xi } \right)C_{0} (2i\xi x)d\xi } , $$

where $C_{n} (x) = \sum\nolimits_{r = 0}^{\infty } {\frac{{( - x)^{r} }}{r!(r + n)!}}$, ($n \in {\rm N}_{0}$) is the Bessel–Tricomi function (Watson 1944), related to the Bessel–Write function $C_{n} (x) = W_{n} ( - x,1)$ and to the commonly known cylindric Bessel functions $C_{n} (x) = x^{ - n/2} J_{n} (2\sqrt x )$. Thus, the solution (81) with the initial condition $g(x) = W_{0} ( - x^{2} ,2)$ is explicitly determined by the formulae (88) and (90).

#### Heat diffusion type equations

Let us consider the following generalized heat type equation with the linear term

91 $$ \partial_{t} F(x,t) = \alpha \partial_{x}^{2} F(x,t) + \beta xF(x,t) $$

with the initial condition $F(x,0) = f(x)$. The solution of Eq. (91) reads (for example, see Zhukovsky and Dattoli 2011 for $\alpha = 1$):

92 $$ F(x,t) = e^{\varPhi (x,t;\beta )} \hat{\varTheta }\hat{S}f\left( x \right) = e^{\varPhi (x,t;\beta )} f(x + \beta t^{2} ,t), $$

where $\hat{\varTheta } = e^{{ab\partial_{x} }}$, $\hat{S} = e^{{a\,\partial_{x}^{2} }}$, $\varPhi (x,t;\beta ) = \frac{1}{3}ab^{2} + bx$, $a = \alpha t,\;b = \beta t$. The solution (92) consists in the action of the evolution operator on the initial condition $F(x,0) = f(x)$, which is transformed by $\hat{\bar{S}}$ and $\hat{\bar{\varTheta }}$. Let us choose now the initial condition $f\left( x \right) = x^{n}$ for the heat diffusion type Eq. (91). Then, upon the action of the $\hat{\bar{S}}$ operator on it and according to the operational definition of the Hermite polynomials (39) $e^{{a\partial_{x} }} x^{n} = H_{n} \left( {x,a} \right)$, we obtain the solution $F\left( x \right) \propto H_{n} \left( {x + ab,a} \right)$, and we end up with

93 $$ F\left( {x,t} \right) = e^{\varPhi } H_{n} \left( {x + \alpha \beta t^{2} ,\alpha t} \right). $$

Let us now consider the following equation:

94 $$ \partial_{t} F\left( {x,t} \right) = \partial_{x}^{2} F\left( {x,t} \right) + 2\delta \partial_{x} F\left( {x,t} \right) + \beta xF\left( {x,t} \right) + \gamma F\left( {x,t} \right),\quad F\left( {x,0} \right) = f\left( x \right). $$

It can be viewed as Eq. (91) where we substituted $\partial_{x} \to \partial_{x} + \delta$ and set $\alpha = 1$. Distinguishing the perfect square of the operator $\partial_{x} + \delta$, we can search the desired solution of the Eq. (94) in the following form:

95 $$ F\left( {x,t} \right) = \exp \left( {t\left( {\gamma - \delta^{2} } \right) - \delta x} \right)G\left( {x,t} \right), $$

where $G\left( {x,t} \right)$ satisfies the Eq. (91) for $G$ with the initial condition $G\left( {x,0} \right) = g\left( x \right)$, $g\left( x \right) = \exp (\delta  x)f\left( x \right)$. Let us choose the initial condition for (94), for example, in the form of the powers of *x*: $f\left( x \right) = x^{k}$. Then $g\left( x \right) = x^{k} e^{\delta x}$ and we have in fact Eq. (91) for $G$: $\partial_{t} G = (\partial_{x}^{2} + \beta x)G$. Upon the action of the $\hat{\bar{S}}$ operator on it and due to the operational rule (52) we obtain $\hat{S}g\left( x \right) = \exp \left( {\delta \left( {x + \delta a} \right)} \right)H_{k} \left( {x + 2\delta a,a} \right) = g\left( {x,t} \right)$. The consequent action of the translation operator $\hat{\varTheta }$ yields the shift along the *x* argument and, thus, the solution of Eq. (91) for $G$, taken $G\left( {x,0} \right) = g\left( x \right)$, has the following form:

96 $$ G\left( {x,t} \right) = e^{{\Phi  + \Delta _{1} }} H_{k} \left( {x + \left( {\alpha \delta ^{2} /\beta } \right)\left( {2t\beta /\delta  + t^{2} \left( {\beta /\delta } \right)^{2} } \right),\alpha t} \right), $$

where $\Delta _{1} = \delta \left( {x + \delta \alpha t + \alpha \beta t^{2} } \right)$. For $\delta = 0$ it immediately returns the result (93). Studying the evolution at prolonged times, such that $t >  > \delta /\beta$ notice that $\Delta _{1} < < \varPhi$ and, provided at so long times the special condition $x >  > \delta ^{2} \alpha /\beta$ is also fulfilled, that is the coordinate travel is limited, we end up with the separation of the dependence of the solution on time and coordinate. The coordinate dependence is contained in the exponential factor $\exp \left( {\beta tx} \right)$, while the time is contained in this factor as well as in the Hermite polynomial arguments $H_{k} \left( {2\delta \alpha t + \alpha \beta t^{2} ,\alpha t} \right)$. For the short times of the evolution of the system, such that $t <  < \delta /\beta$ the phase approximately reads $\varPhi +\Delta _{1} \cong x\delta + \alpha \delta^{2} t$ and the Hermite polynomials depend on both coordinate and time: $H_{k} \left( {x,\alpha t} \right)$. Thus, for relatively short times we have the solution approximated by $\left. {G\left( {x,t} \right)} \right|_{t \to 0} = e^{{x\delta + \alpha \delta^{2} t}} H_{k} \left( {x,\alpha t} \right)$ and for infinitely short times $\alpha t \to 0$ the Hermite polynomials become $H_{k} \left( {x,0} \right) = x^{k}$, which is perfect agreement with our initial condition $g\left( x \right) = x^{k} e^{{\delta  x}}$. The desired solution of the Eq. (94) follows immediately upon the assumption of $\alpha = 1$ in (96) with different phase $\Delta _{2} = t\gamma + t^{2} \delta \beta$:

97 $$ F\left( {x,t} \right) = e^{{\varPhi + \varDelta_{2} }} H_{k} \left( {x + 2t\delta + t^{2} \beta ,t} \right). $$

The two-dimensional heat diffusion type equation with the linear terms

98 $$ \partial_{t} F\left( {x,y,t} \right) = \left\{ {\left( {\alpha \partial_{x}^{2} + \beta \partial_{x} \partial_{y} + \gamma \partial_{y}^{2} } \right) + bx + cy} \right\}F\left( {x,y,t} \right),\quad \hbox{min} \left( {\alpha ,\beta ,\gamma } \right) > 0, $$

and the initial condition $F\left( {x,y,0} \right) = f\left( {x,y} \right)$ can be solved following (Zhukovsky 2014) or with the help of the Baker–Campbell–Hausdorf formula $\exp \left( {\hat{A} + \hat{B}} \right) = \exp \left( { - [\hat{A},\hat{B}]{\text{ /}}2} \right)\exp \left( {\hat{A}} \right)\exp \left( {\hat{B}} \right)$. In complete analogy with the one-dimensional case, we obtain the solution of the two-dimensional heat conduction equation with lineal terms (98) in the following form:

99 $$ F(x,y,t) = e^{\varPsi } \hat{\varTheta }_{x} \hat{\varTheta }_{y} \hat{\rm E}f\left( {x,y} \right) \propto f \left( {x + t^{2} \left( {\alpha b + \beta c/2} \right),y + t^{2} \left( {\gamma c + \beta b/2} \right),t} \right).  $$

where $\varPsi = (\alpha b^{2}  + \gamma c^{2}  + \beta bc)t^{3} /3 + t(bx + cy)$ is the phase, $\hat{\varTheta }_{x} = e^{{\,t^{2} \left( {\alpha b + \beta c/2} \right)\partial_{x} }}$ and $\hat{\varTheta }_{y} = e^{{t^{2} \left( {\gamma c + \beta b/2} \right)\partial_{y} }}$ are the diffusion operators for each of the two coordinates, and

100 $$ \hat{\rm E} = \exp \left[ {t\left( {\alpha \partial_{x}^{2} + \beta \partial_{x} \partial_{y} + \gamma \partial_{y}^{2} } \right)} \right] $$

is the two-dimensional analogue of heat diffusion operator $\hat{S}$ (25). The explicit double integral form of the operator $\hat{\rm E}$ was obtained in Dattoli et al. (2006) and it is the Gauss type integral, which we omit here for the sake of conciseness. It is easy to demonstrate that in the case of $\beta = 0$ the heat diffusion is executed by the one-dimensional operators (25) $\hat{S}_{x} \hat{S}_{y}$ instead of the more general operator $\hat{\rm E}$. The solution in this case reads as follows:

101 $$ F(x,y,t) = e^{\varPsi } \hat{\varTheta }_{x} \hat{\varTheta }_{y} \hat{S}_{x} \hat{S}_{y} f\left( {x,y} \right) \propto f\left( {x + t^{2} \alpha b,y + t^{2} \gamma c,t} \right). $$

Note, that the operational definition (100) allows for the direct computation of the result in many cases, avoiding the necessity to calculate the double integral for the operator $\hat{\rm E}$. Let us, for example, choose the initial function in the form of the powers of *x* and *y*: $f\left( {x,y} \right) = x^{m} y^{n}$. Then, according to the operational definition of the Hermite polynomials of four variables and two indices $H_{m,n} \left( {\left. {x,t\alpha ,y,t\gamma } \right|\beta } \right)$ (see, for example, Erdélyi et al. 1953; Dattoli et al. 2006, 2007) we obtain

102 $$ \hat{\rm E}\left\{ {x^{m} y^{n} } \right\} = H_{m,n} \left( {\left. {x,t\alpha ,y,t\gamma } \right|t\beta } \right), $$

where $H_{m,n} \left( {\left. {x,t\alpha ,y,t\gamma } \right|\beta } \right)$ are the above mentioned Hermite polynomials with the following generating exponent:

103 $$ \sum\limits_{m,n}^{\infty } {\frac{{u^{m} v^{n} }}{m!n!}H_{m,n} \left( {\left. {x,\alpha ,y,\gamma } \right|\beta } \right)} = \exp \left( {xu + \alpha u^{2} + yv + \gamma v^{2} + \beta uv} \right). $$

The presentation of $H_{m,n} \left( {\left. {x,t\alpha ,y,t\gamma } \right|\beta } \right)$ in the form of sums (see Erdélyi et al. 1953) of the two-variable Hermite polynomials $H_{m} \left( {x,y} \right)$, defined in (39), reads as follows:

104 $$ H_{m,n} \left( {\left. {x,\alpha ;y,\gamma } \right|\beta } \right) = m!n!\sum\limits_{s = 0}^{{\rm{min} \left( {m,n} \right)}} {\frac{{\beta^{s} }}{s!(m - s)!(n - s)!}H_{m - s} \left( {x,\alpha } \right)H_{n - s} \left( {y,\gamma } \right)} . $$

The action of the translation operators $\hat{\varTheta }_{x} \hat{\varTheta }_{y}$ on the Hermite polynomials $H_{m,n} \left( {\left. {x,t\alpha ,y,t\gamma } \right|\beta } \right)$ yields the solution of the two-dimensional heat type equation with the linear terms (98) and with the initial condition in the form of powers $f\left( {x,y} \right) = x^{m} y^{n}$:

105 $$ F\left( {x,t} \right) = e^{\varPsi } H_{m,n}  \left( {\left. {x + t^{2} \left( {\alpha b + \beta c/2} \right),t\alpha ;y + t^{2} \left( {\gamma c + \beta b/2} \right),t\gamma } \right|t\beta } \right) . $$

It appears evident that the obtained solution (105) of the two-dimensional heat conduction problem (98) represents a direct generalization of the solution (93) for the one-dimensional heat conduction analog (91).

### Operational solution of some second order of time partial differential equations

The operational method for solution of differential equations can be successfully applied for the second order of time partial differential equations too. Let us consider the equations of the following type:

106 $$ \left( {\frac{{\partial^{2} }}{{\partial t^{2} }} + \varepsilon \frac{\partial }{\partial t}} \right)F\left( {x,t} \right) = \hat{D}\left( x \right)F\left( {x,t} \right), $$

where $\hat{D}\left( x \right)$ is a differential operator, acting over the coordinate, such as, for example, the heat diffusion operator $\partial_{x}^{2}$ or the Laguerre derivative $_{L} D_{x}$ or any other. General solution of the Eq. (106) reads as follows:

107 $$ F\left( {x,t} \right) = e^{{ - \frac{t\varepsilon }{2}}} \left( {e^{{ - \frac{t}{2}\sqrt {\varepsilon^{2} + 4\hat{D}\left( x \right)} }} C_{1} \left( x \right) + e^{{\frac{t}{2}\sqrt {\varepsilon^{2} + 4\hat{D}\left( x \right)} }} C_{2} \left( x \right)} \right), $$

where $C_{1,2} \left( x \right)$ are to be determined from the initial conditions. Suppose the initial condition $F\left( {x,0} \right) = f\left( x \right)$ is given and, for example, we can require for the second order Eq. (106) that its solution converges at infinite time $t \to \infty$. Other initial and boundary conditions are, of course, possible, but they will be considered elsewhere. Our choice sets $C_{2} \left( x \right) = 0$ and the remaining branch of the solution is subject to the Laplace transforms

108 $$ e^{ - t\sqrt V } = \frac{t}{2\sqrt \pi }\int\limits_{0}^{\infty } {\frac{d\xi }{\xi \sqrt \xi }e^{{ - \frac{{t^{2} }}{4\xi } - \xi V}} } ,\quad t > 0. $$

Thus, we obtain for the Eq. (106) the following fading at infinite times solution:

109 $$ F\left( {x,t} \right) = e^{{ - \frac{\varepsilon t}{2}}} \frac{t}{4\sqrt \pi }\int\limits_{0}^{\infty } {\frac{d\xi }{\xi \sqrt \xi }e^{{ - \frac{{t^{2} }}{4\xi } - \xi \varepsilon^{2} }} \exp \left( { - 4\xi \hat{D}\left( x \right)} \right)f\left( x \right)} , $$

provided the integral converges. The particular form of the solution depends on the operator $\hat{D}\left( x \right)$ and on the initial function $f\left( x \right)$.

Let us first consider the following equation:

110 $$ \frac{{\partial^{2} }}{{\partial t^{2} }}F\left( {x,t} \right) = \hat{D}\left( x \right)F\left( {x,t} \right),\quad F\left( {x,0} \right) = f\left( x \right),F\left( {x,\infty } \right) < \infty . $$

Then (107) reduces to $F(x,t) = \left( {e^{{ - t\sqrt {\hat{D}\left( x \right)} }} C_{1} \left( x \right) + e^{{t\sqrt {\hat{D}\left( x \right)} }} C_{2} \left( x \right)} \right)$. For $\hat{D}\left( x \right) = {}_{L}D_{x}$ it becomes

111 $$ \frac{{\partial^{2} }}{{\partial t^{2} }}F\left( {x,t} \right) = \partial_{x} x \partial_{x} F\left( {x,t} \right). $$

We now make use of the identity

112 $$ \frac{t}{2}\int\limits_{0}^{\infty } {\frac{{u^{m} du}}{u\sqrt u }\exp \left( { - \frac{{t^{2} }}{4u}} \right)} = 4^{ - m} t^{2m} \varGamma \left( {\frac{1}{2} - m} \right), $$

which yields the following bounded at infinite times solution of Eq. (111) for the initial function $f\left( x \right) = x^{n}$ in terms of the Gegenbauer polynomial $C_{n}^{k}$ and of the gamma function Γ:

113 $$ F\left( {x,t} \right) = \frac{{n!}}{{\sqrt \pi  }}\sum\limits_{{r = 0}}^{n} {\frac{{\left( { - x} \right)^{r} 4^{{ - \left( {n - r} \right)}} t^{{2\left( {n - r} \right)}} \Gamma\left[ {1/2 - \left( {n - r} \right)} \right]}}{{\left( {n - r} \right)!\left( {r!} \right)^{2} }}}  = n!\frac{{\left( { - x} \right)^{n} C_{{2n}}^{{\left( { - 2n} \right)}} \left( {\frac{t}{{2\sqrt x }}} \right)}}{{\Gamma \left( {1 + 2n} \right)}}.  $$

For example, for $m = 2$ (113) immediately reduces to $\left. {F\left( {x,t} \right)} \right|_{{n = 2}}  = \left( {t^{4}  + 12t^{2} x + 6x^{2} } \right){\text{ /}}12$.

Let us consider the Black–Scholes type differential operator in the r.h.s. of the second order differential equation, namely

114 $$ \left( {\frac{{\partial^{2} }}{{\partial t^{2} }} + \varepsilon \frac{\partial }{\partial t}} \right)G\left( {x,t} \right) = \left( {x^{2} \frac{{\partial^{2} }}{{\partial x^{2} }} + \lambda x\frac{\partial }{\partial x} - \mu  } \right)G\left( {x,t} \right),\quad G\left( {x,0} \right) = g\left( x \right),G\left( {x,\infty } \right) < \infty . $$

Distinguishing the full square of the operator $\bar{D} = x\partial_{x}$, we have to compute the result of the action of the exponential operator $\exp \left[ { - 4\xi \left( {\left( {\bar{D} + \lambda /2} \right)^{2}  - \nu } \right)} \right]g\left( x \right)$, where $\nu  = \mu  + (\lambda /2)^{2}$. To achieve it, we exploit the operational identity (16), applying it to the exponential $\exp \left[ {\left( {\bar{D} + \lambda /2} \right)^{2} } \right]$: $e^{{ - 4\xi \left( {\bar{D} + \lambda /2} \right)^{2} }}  = \int_{{ - \infty }}^{\infty } {\exp \left[ { - u^{2}  + 2iu\lambda \sqrt \xi   + 4iu\sqrt \xi  \bar{D}} \right]du/\sqrt \pi }$. Upon the action on $g\left( x \right)$, we obtain $\exp \left[ {4iu\sqrt \xi x\partial_{x} } \right]g\left( x \right) = g\left( {e^{4iu\sqrt \xi } x} \right)$, which yields the function

115 $$g\left( {x,\xi } \right) = e^{{ - 4\xi \left( {\bar{D} + \lambda /2} \right)^{2} }} f\left( x \right) = \frac{1}{{\sqrt \pi  }}\int\limits_{{ - \infty }}^{\infty } {e^{{ - u^{2}  + 2iu\lambda \sqrt \xi  }} g\left( {e^{{4iu\sqrt \xi  }} x} \right)du},$$

and the solution of Eq. (114) now takes the following form:

116 $$ G\left( {x,t} \right) = e^{{ - t\varepsilon /2}} \frac{t}{{4\sqrt \pi  }}\int\limits_{0}^{\infty } {\frac{{d\xi }}{{\xi \sqrt \xi  }}\exp \left[ { - \frac{{t^{2} }}{{16\xi }} - \varepsilon ^{2} \xi  + 4\xi \nu } \right]} g\left( {x,\xi } \right). $$

Eventually, let us consider the following rather complicated differential equation of second order of time and coordinate:

117 $$ \left( {\frac{{\partial^{2} }}{{\partial t^{2} }} + \varepsilon \frac{\partial }{\partial t}} \right)F\left( {x,t} \right) =    \left[ {x^{2}  \frac{{\partial^{2} }}{{\partial x^{2} }} + 2\alpha x^{2} \frac{\partial }{\partial x} + \lambda  x\frac{\partial }{\partial x} + (\alpha x)^{2} - \mu  } \right]F\left( {x,t} \right). $$

with the initial condition $F\left( {x,0} \right) = f\left( x \right)$ and we seek non-diverging at infinite times solution $F\left( {x,\infty } \right) < \infty$. The solution arises directly from (116), (115) and from (114). Indeed, by noting that Eq. (117) is in fact Eq. (114) with $\partial_{x} + \alpha$ instead of $\partial_{x}$, we write our solution $F(x,t) = e^{ - \alpha x} G(x,t)$, where $G\left( {x,t} \right)$ satisfies Eq. (114) with the initial condition $g(x) = G(x,0) = e^{\alpha x} F(x,0) = e^{\alpha x} f(x)$, and we demand $F\left( {x,\infty } \right) < \infty$ and $G\left( {x,\infty } \right) < \infty$ respectively. Now the expression (116) for $G\left( {x,t} \right)$ with account for (115), where $g(x) = e^{\alpha x} f(x)$, provide our solution $F(x,t) = e^{ - \alpha x} G(x,t)$. Consider the simple example of the initial function $f\left( x \right) = e^{ - \alpha x} x^{n}$, which illustrates the above-sketched technique. It returns $g\left( x \right) = x^{n}$ and we easily obtain upon the integration over $du$ and $d\xi$ the function

118 $$G\left( {x,t} \right) = x^{n} \exp \left[ { - t/2\left( {\varepsilon  + \sqrt {\varepsilon ^{2}  + 4\left( {n\left( {n - 1 + \lambda } \right)} \right) - \mu } } \right)} \right].$$

It, in turn, yields the desired solution $\left. {F\left( {x,t} \right)} \right|_{t \to \infty } < \infty$ of (117) with $f\left( x \right) = e^{ - \alpha x} x^{n}$ as follows:

119 $$ F\left( {x,t} \right) = x^{n} \exp \left[ { - \alpha x - t/2\left( {\varepsilon  + \sqrt {\varepsilon ^{2}  + 4\left( {n\left( {n - 1 + \lambda } \right)} \right) - \mu } } \right)} \right]. $$

The solution for the particular case $\varepsilon = 0$ reads as follows: $\left. {F\left( {x,t} \right)} \right|_{{\varepsilon  = 0}}  = x^{n} e^{{ - \alpha x - t\sqrt {n\left( {n - 1 + \lambda } \right) - \mu /4} }}$.

### Hyperbolic heat equation solution

Another example of the operator $\hat{D}\left( x \right)$ in the r.h.s of Eq. (106) is given by the $\partial_{x}^{2}$:

120 $$ \left( {\frac{{\partial^{2} }}{{\partial t^{2} }} + \varepsilon \frac{\partial }{\partial t}} \right)F\left( {x,t} \right) = \alpha \frac{{\partial^{2} }}{{\partial x^{2} }}F\left( {x,t} \right),\quad F\left( {x,0} \right) = f\left( x \right),F\left( {x,\infty } \right) < \infty , $$

which is one-dimensional case of the Cattaneo’s hyperbolic heat conduction equation (Cattaneo 1958)

121 $$ (\tau \partial_{t}^{2} + \partial_{t} )T = k_{T} \nabla^{2} T, $$

where $\tau  = 1/\varepsilon$ is an intrinsic thermal property of the media, characterizing the time needed for the initiation of a heat flow after a temperature gradient appears at the boundary of the domain, and $k_{T}  = \alpha /\varepsilon$ denotes heat diffusivity. The time $\tau  = 1/\varepsilon$ is often related to the speed of the second sound *C* in media ($\tau  = k_{T} /C^{2}$); $\sqrt {k_{T} /\tau }  = C$ represents a velocity like quantity, associated with the speed of the heat wave in the medium, which characterizes the thermal wave propagation the same way as the diffusion behaviour is characterized by the diffusivity. Equation (121) is the simplest model of the second sound phenomenon observed first in liquid Helium (Peshkov 1944) and then also in solid crystals (Ackerman and Overton 1969). To solve it we have to compute the result of the action of the operator $\hat{S}$: $e^{{ - 4\alpha \xi \partial_{x}^{2} }} f\left( x \right)$. The fading at infinite time solution for the initial function $f\left( x \right)$ follows directly from (109):

122 $$ F\left( {x,t} \right) = e^{{ - \frac{\varepsilon t}{2}}} \frac{t}{4\sqrt \pi }\int\limits_{0}^{\infty } {\frac{d\xi }{\xi \sqrt \xi }e^{{ - \frac{{t^{2} }}{16\xi } - \varepsilon^{2} \xi }} \hat{S}f\left( x \right)} ,\quad \hat{S}f\left( x \right) = e^{{ - 4\alpha \xi \partial_{x}^{2} }} f\left( x \right). $$

The action of the operator $\hat{S}$ can be accomplished with the help of the identity (16):

123 $$ F\left( {x,t} \right) = e^{{ - \frac{\varepsilon t}{2}}} \frac{t}{4\sqrt \pi }\int\limits_{0}^{\infty } {\frac{du}{u\sqrt u }e^{{ - \frac{{t^{2} }}{16u} - \varepsilon^{2} u}} \frac{1}{\sqrt \pi }\int\limits_{ - \infty }^{\infty } {e^{{ - v^{2} }} f\left( {x + 4iv\sqrt {u\alpha } } \right)dv} } , $$

Let us consider the initial monomial $f\left( x \right) = x^{n}$, for which we obtain the Hermite polynomials upon the action of the operator $\hat{S}$, as follows from the operational definition (39) with account for (41). Thus, the solution of Eq. (120) takes the following explicit form:

124 $$  \begin{aligned}   \left. {F\left( {x,t} \right)} \right|_{{F\left( {x,0} \right) = x^{n} }}  &  = e^{{ - t\varepsilon /2}} \frac{{t\left( { - i} \right)^{n} \left( { - 4\alpha } \right)^{{n/2}} }}{{4\sqrt \pi  }}\int\limits_{0}^{\infty } {u^{{\frac{{n - 3}}{2}}} H_{m} \left( {x/4\sqrt {\alpha u} } \right)\exp \left[ { - \frac{{t^{2} }}{{16u}} - u\varepsilon ^{2} } \right]}  \\     &  = e^{{ - \frac{t}{{2\tau }}}} \sqrt {\frac{t}{{\pi \tau }}} n!\sum\limits_{{r = 0}}^{{\left[ {n/2} \right]}} {\frac{{\left( x \right)^{{n - 2r}} }}{{\left( {n - r} \right)!r!}}( - tk_{T} )^{r} K_{{\frac{1}{2} - r}} \left( {\frac{t}{{2\tau }}} \right)}  \\  \end{aligned} $$

Let us now consider the initial function $F\left( {x,0} \right) = e^{\gamma x} x^{n}$. We make use of the operational identity (52) to obtain the following integral form of the solution:

125 $$ \left. {F\left( {x,t} \right)} \right|_{{F\left( {x,0} \right) = x^{n} e^{\gamma x} }} = \frac{{te^{{ - \frac{t\varepsilon }{2} + \gamma x}} }}{4\sqrt \pi }\int\limits_{0}^{\infty } {\frac{{H_{n} \left( {x - 8\gamma \alpha u, - 4\alpha u} \right)}}{u\sqrt u }\exp \left[ { - \frac{{t^{2} }}{16u} - u\left( {\varepsilon^{2} + 4\alpha \gamma^{2} } \right)} \right]} du. $$

For brevity we omit here the result of the above integration, which is rather cumbersome. In the particular case of given *n* and *γ*, for the example for $n = - \gamma = 1$, we obtain

126 $$\left. {F\left( {x,t} \right)} \right|_{{f\left( x \right) = xe^{{ - x}} }}= e^{{ - x - \frac{{t\varepsilon }}{2} - \frac{t}{2}\sqrt {4\alpha  + \varepsilon ^{2} } }} \left( {x \,+ } \,\frac{{2t\alpha }}{{\sqrt{4\alpha  + \varepsilon ^{2} }}} \right)= e^{{ - x - \frac{t}{{2\tau }}\left( {1 + \sqrt {1 + 4\tau k_{T} } } \right)}} \left( {x \,+ } \,\frac{{2tk_{T} }}{{(1 + 4\tau k_{T} )^{{1/2}} }} \right)$$

**Fig. 1:**
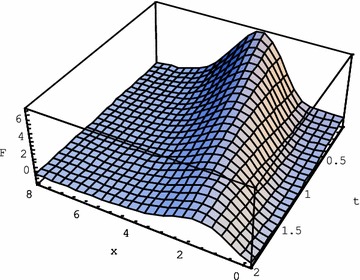
Solution (126) of the Eq. (121) for $k_{T} = 1,\,\tau = 1$ for the initial function $F\left( {x,0} \right) = e^{ - 3x} x^{10}$

The example of $\left. {F\left( {x,t} \right)} \right|_{{f\left( {0,t} \right) = x^{10} e^{ - 3x} }}$ for $n = 10,\;\gamma = - 3$ is more demonstrative for graphical presentation. We omit here the exact formula for this solution for brevity and present its behaviour for $\alpha = \varepsilon = 1$, i.e. $k_{T} = 1,\;\tau = 1$, in Fig. 1. Observe fading wave propagation.

**Fig. 2:**
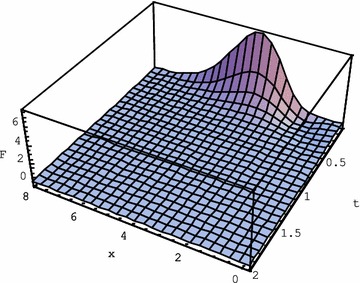
Solution (126) of the Eq. (121) for $k_{T} = 1,\,\tau = 0.1$ for the initial function $F\left( {x,0} \right) = e^{ - 3x} x^{10}$

By choosing $\alpha = \varepsilon = 10$ in (120), i.e. $k_{T} = 1,\;\tau = 0.1$, we reduce the effect of the second time derivative in the equation, maintaining the heat conductivity unchanged. In this case the fading of the solution happens earlier, as seen in Fig. 2. The diffusive character of the heat conduction in this case prevails over the wave-like propagation process.

**Fig. 3:**
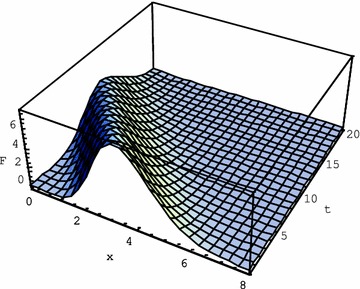
Solution (126) of the Eq. (121) for $k_{T} = 1,\,\tau = 10$ for the initial function $F\left( {x,0} \right) = e^{ - 3x} x^{10}$

Contrary to this case, the choice of $\alpha = \varepsilon = 0.1$ in (120), i.e. $k_{T} = 1,\;\tau = 10$, underlines the wave-like propagation of the initial function in Fig. 3.

Non-Fourier diffusive, wave-like heat propagation in Cattaneo’s model (120), (121) had some success in the description of second sound. However, it did not agree with the experimental observations and it was superseded by other, more adequate heat propagation models, which included additional terms in the hyperbolic equation to describe the whole complex of phenomena. We will obtain analytical solutions for them in forthcoming publications.

## Results and conclusions

We advocated operational method for solution of linear differential equations. We use inverse differential operators; it allows direct and straightforward computation of solutions in the framework of operational calculus. The obtained solutions contain consequent action of operators of heat conduction and shift, involving common and Laguerre derivative with exponential and power factors. Their action can be expressed via Gauss type integrals and shift of arguments. Using operational definitions of Hermite and Laguerre orthogonal polynomial families, we executed direct operational transforms over them, consisting in shift and factorization. Combined where necessary with integral transforms, it yielded solutions of relatively complicated linear differential equations of several types.

In particular, we have obtained explicit exact solutions for some ordinary differential equations of non-integer dimension, involving shifted derivatives. The particular solution of equation $\psi^{ - 1} (D) = \left( {\beta^{2} - (D + \alpha )^{2} } \right)^{ - \nu } f(x)$ for any Real ν is given by the integral of the weighted consequent action of operators of heat propagation $\hat{S}$ and translation $\hat{\varTheta }$ on the function $f(x)$. We wrote it as a convolution transform $\phi \left( {x,\tau } \right) = G\left( {x,\tau } \right) \cdot f\left( \eta \right)$ or $\phi = \int\nolimits_{ - \infty }^{\infty } {G\left( {x - \eta } \right)f\left( \eta \right)d\eta }$ with the kernel, equal to the Gauss frequency function. The examples of Gaussian $f(x) = e^{{ - x^{2} }}$ and monomial functions $f(x) = x^{k}$ were demonstrated by explicit solutions in terms of integrals and series of Hermite polynomials. Operational solutions for equations, involving Laguerre derivatives: $(x\partial^{2}_{x} + \left( {\alpha + 1} \right)\partial_{x} )^{\nu } F(x) = f(x)$ were obtained. Examples of the exponential $f(x) = \exp ( - \gamma x)$, of the monomial and of Bessel–Wright functions were demonstrated. By using operational technique we immediately write their explicit solutions; they involve integrals and generalized Laguerre polynomials.

We obtained solutions for several types of partial differential equations. In particular, extended Black–Scholes equation was solved. Moreover, generalized form of Black–Scholes type equation with Laguerre derivative ${}_{L}D_{x} = \partial_{x} x \partial_{x}$ was solved operationally. The example of a monomial initial function yields the explicit solution with series of gamma function and hypergeometric function. For initial Bessel–Wright function the solution of the Black–Scholes equation with Laguerre derivative is given by integrals of Bessel–Tricomi function.

Extended forms of heat diffusion equation were solved. Their operational solution readily yields explicit forms upon consequent action of operators of shift and heat diffusion on the initial function. Examples of initial functions $g\left( x \right) = x^{k} e^{\delta x}$ and $f\left( {x,y} \right) = x^{m} y^{n}$ produce Hermite polynomials and their generalized forms with four variables and two indices: $H_{m,n} \left( {\left. {x,t\alpha ,y,t\gamma } \right|\beta } \right)$. Two-dimensional heat diffusion type equation with the linear terms was solved. Its operational solution consists in the action of the generalized two-dimensional analogue of heat diffusion operator and respective coordinate shifts with a phase factor.

Operational solutions of a number of hyperbolic equations with regular and Laguerre coordinate derivatives were obtained. For the second order of time hyperbolic equations with Laguerre derivatives of the 1st and 2nd order we obtained explicit solutions in terms of elementary functions, Gegenbauer polynomials and gamma function for the initial monomial $f\left( x \right) = x^{n}$ and for the exponential $f\left( x \right) = x^{m} e^{ - \alpha x}$. Hyperbolic heat equation was thoroughly explored with the help of operational method. Explicit solution for $f\left( x \right) = x^{m} e^{ - \alpha x}$ was obtained in elementary functions. The role of various equation terms in the behaviour of the solution was elucidated. Maintaining the heat conductivity unchanged, we underlined the role of the second time derivative. Fading of the wave propagation happens earlier, if we reduce the effect of the second time derivative in the equation, choosing its coefficient small with respect to others: $k_{T} = 1,\;\tau = 0.1$. In this case the diffusive character of the heat conduction prevails over the wave-like propagation process. On the contrary, high value of the second time derivative term in (120): $k_{T} = 1,\,\tau = 10$, underlines the wave-like propagation with very little fading as follows from the comparison of Figs. 1, 2 and 3.

Thus, operational approach allowed for easy and straightforward solution of differential equations and relevant physical problems, such as modified Fourier heat diffusion in three dimensions, Cattaneo heat propagation, Laguerre type diffusion, evolution of a system, obeying Black–Scholes type equations, common in financial studies. Operational method has obvious advantages, respectively to other methods: it is universal, applies to ordinary and partial linear DE and non-integer DE, the solutions are obtained readily; they are light computationally and have transparent meaning. The effect of each term in the initial equation on the solution is distinguished. The validity of the obtained solutions was verified by direct substitution in the solved equations. The solutions in form of integrals contain consequent action of operators of heat conduction and shift with exponential and power factors. The considered examples of solutions of the hyperbolic and Fourier heat equations with common and Laguerre derivatives for given initial functions contain integrals and series of Hermite polynomials; explicit solutions, such as (96), (97), (105), (113), (119), (126) do not possess critical or singular coordinate points. For the second order of time differential Eqs. (106) with ordinary and Laguerre derivatives we obtained strictly bounded solutions. Rigorous investigation of stability of all of the obtained solutions for different initial functions, including fractional differential equations and employing Lyapunov methods, will constitute a stand-alone study in a forthcoming dedicated publication.

In conclusion we would like to note, that our results, being exact, can represent a benchmark for numerical solutions. These latter can, perhaps, cover more extensions, but should reduce to our results in the limiting cases. Application of our study for more complicated equations, describing non-Fourier heat propagation by ballistic heat transfer and other equations, modelling physical processes, will be also made in forthcoming publications.
